# Prognostic Value of UBE2T and Its Correlation with Immune Infiltrates in Lung Adenocarcinoma

**DOI:** 10.1155/2022/5244820

**Published:** 2022-09-20

**Authors:** Feng Xu, Na Xiong, Yuhong Yuan, Jun Liu

**Affiliations:** ^1^Department of Respiratory Diseases, Qingdao Chengyang District People's Hospital, Qingdao, China; ^2^Critical Care Medicine, Qingdao Eighth People's Hospital, Qingdao, China; ^3^Department of Pharmacy Qingdao Chengyang District People's Hospital, Qingdao, China; ^4^Department of General Surgery, Gaomi Hospital of Traditional Chinese Medicine, Gaomi, China

## Abstract

Non-small cell lung cancer has a subtype with a high morbidity and mortality rate called lung adenocarcinoma (LUAD). It is critical to locate reliable prognostic biomarkers for LUAD at this time. Ubiquitin-conjugating enzyme E2T (UBE2T) has been found in numerous malignancies; however, its expression level and potential functions in LUAD are not completely understood at this time. A differentially expressed gene (DEG) screening method was used to identify genes that were expressed differently in 516 samples from LUAD and 59 samples from TCGA datasets. Clinicopathological markers were correlated with UBE2T expression. Using the Kaplan–Meier plotter database, UBE2T was evaluated for its prognostic value in the context of LUAD. In order to examine the importance of independent prognostic factors, both univariable and multivariable Cox regression models were applied. TIMER and CIBERSORT were utilized in order to investigate the connection that exists between UBE2T expression and tumor-infiltrating immune cells. This study collected 578 DEGs in total, as follows: 171 genes were significantly increased, while 408 genes were significantly decreased. We identified 9 survival-related DEGs in LUAD, including ASF1B, CA9, CCNB2, CCNE1, RRM2, SAPCD2, TCN1, TPX2, and UBE2T. Our attention focused on UBE2T, which was highly expressed in LUAD. A correlation was also found between high UBE2T expression and gender, age, advanced clinical stage, and decreased overall survival. In addition, multivariate analysis demonstrated UBE2T expression to be a significant independent diagnostic factor for patients suffering from LUAD. UBE2T was positively correlated with resting T cell CD4+ memory, myeloid dendritic cell resting, mast cell activated, macrophage M2, and B cell plasma, whereas it was negatively correlated with resting T cell CD4+ memory, MDC resting, MDC activated, macrophage M2, and B cell plasma. Overall, high expression levels of UBE2T correlated with poor overall survival in patients with LUAD, and UBE2T was an independent predictor involved in immune infiltration of LUAD. These findings offer fresh perspectives that contribute to our comprehension of the evolution of LUAD.

## 1. Introduction

Lung cancer is considered to be one of the most common malignant tumors all over the world [1, 2]. It has virtually reached the position of being the first major contributor to death among those living in China's urban areas [3, 4]. The majority of lung malignancies, approximately 70–80 percent, are diagnosed as non-small cell lung cancer (NSCLC) [5, 6]. Lung adenocarcinoma is a main subtype of NSCLC and is often diagnosed at an advanced disease stage [7]. Early surgical resection is currently the recommended course of treatment for patients diagnosed with LUAD. Following the completion of any necessary surgical procedures, the patient will undergo further chemotherapy to further increase their chances of survival [8, 9]. However, half of all people who have LUAD will suffer a relapse at some point and will ultimately pass away as a result of the disease's return. A reliable method of predicting patient survival status is needed in order to facilitate the diagnosis of early-stage LUAD and to provide patients with reasonable treatment regimens without wasting medical resources or delaying their recovery.

There has been a shift in the therapy paradigm for LUAD over the past few years due to the use of immunotherapies for the therapy of patients suffering from LUAD [10, 11]. Since January 2015, there have been substantial advancements made in cancer immunotherapy [12]. Inhibiting programmed cell death protein 1 is successful in treating Hodgkin's lymphoma, generating optimism about its potential to change the way the disease is typically treated [13, 14]. Immunotherapy using inhibitors of programmed death-1, programmable death ligand-1, and cytotoxic T lymphocyte associated antigen-4 has been demonstrated to possess potential anticancer benefits in malignant melanoma [15, 16]. Growing data suggest that tumor-infiltrating immune cells in the tumor microenvironment contribute to tumor development, aggressiveness, and responsiveness to therapy [17, 18]. Growing evidence supporting the idea that cancer lymphocytes, such as cancer macrophages and cancer neutrophils, affect the prognosis and the efficiency of chemotherapy and immunotherapy is also rising [19]. Additionally, it has become more and more common to block immunological checkpoints like PD-1/PD-L1 and CTLA-4 in malignant tumors [20, 21]. The majority of malignancies do not react well to immunotherapy with a single drug because the tumor microenvironment contains immune elements. Clarifying immunogen types of tumor-immune interactions as well as finding new immune-related biomarkers and targeted therapies in LUAD are urgently needed.

The TCGA project, which was completed just recently, includes matched clinical and molecular data of numerous tumors, which makes it possible to conduct a systematic investigation of the impact that single gene expression has on patients' chances of survival. In this study, we aimed to explore novel biomarkers via analyzing TCGA datasets. Using LUAD cohorts-based TCGA datasets, we screened differentially expressed genes (DEGs). We identified a novel LUAD-related gene ubiquitin-conjugating enzyme E2T (UBE2T) which was significantly expressed in LUAD and predicted a poor prognosis. UBE2T plays a significant function in a variety of pathological processes in a manner that is E2-enzyme-dependent. The reason for this is that it belongs to the E2 family of proteins, which are responsible for conjugating ubiquitin to substrates [22]. The expression of UBE2T has been reported to be dysregulated in several tumors, including cancers of the stomach, liver, and esophagus [23–25]. Despite this, there has been no investigation of the prognostic value of UBE2T in LUAD. Based on our findings, a new prognostic biomarker that is involved in the microenvironment of tumors may be developed for LUAD.

## 2. Materials and Methods

### 2.1. Acquiring and Processing Raw Data

Over 10,000 cancer patients whose tumors were classified into one of 33 categories have been assessed and evaluated by the TCGA research network. To obtain transcriptome data of 33 different tumor types, we searched the TCGA database (https://portal.gdc.cancer.gov/). A total of 33 cancer types were studied. They were OV, PAAD, PRAD, READ, SKCM, STAD, TGCT, THCA, THYM, UCEC, and UCS. ACC, BLCA, BRCA, COAD, DLBC, ESCA, GBM, HNSC, KICH, KIRC, KIRP, LAML, LGG, LIHC, and UCS. The full names of all tumors are shown in Table S1.

### 2.2. Genes Differentially Expressed in LUAD Identified

Data from our research were mapped against version 38 (hg38) of the human genome using the STAR2 software. This allowed us to generate data on gene expression. The Sam Tools were utilized in order to identify the mapped reads that had a quality of 10 or higher. The feature count served as the reference transcriptome to define the read counts for each gene. With the aid of the edger package in *R*, differential expression analysis was conducted, and tumor samples were compared with normal samples that were matched to them in order to identify DEGs [26]. Among the genes that were selected for differential expression between tumor and normal samples, their false discovery rates (FDR) are less than 0.05 and their absolute log2 fold changes (log FC) are greater than 4.

### 2.3. An Analysis of the Expression of UBE2T in Pan-Cancers

The TCGA and Genotype Tissue Expression (GTEx) projects provided data on the differential expression of UBE2T between tumor and normal tissue that was matched to a tumor. A tissue bank and data resource called GTEx has been established by the National Institutes of Health Common Fund (https://gtexportal.org). A total of 53 human normal tissues from about 1,000 people were examined for genetic variants, RNA sequencing, and additional molecular traits. We chose log2 (TPM+1) converted expression data for plotting, which was how we chose the parameters.

### 2.4. Infiltration Cells and Their Marker Genes Are Correlated with the UBE2T Expression

Whether UBE2T expression and immune cell presence were correlated was investigated using the Tumor Immune Estimation Resource database (TIMER) [27]. The TIMER database greatly assisted in the evaluation and integration of immune cells for RNA sequencing samples from the TCGA. These immune cells are thought to contain human B cells, human CD4+ T cells, human CD8+ T cells, human macrophages, human neutrophils, and human dendritic cells. The proportional fractions of 22 different immune cell types invading each tumor sample were calculated using the *R* tool CIBERSORT.

### 2.5. An Analysis of the Relationship between UBE2T Gene Expression and Immune Markers

In this research, we examined the relationship between more than 40 immune checkpoint genes and UBE2T expression. The *R* software program “GGplot2” was used to retrieve these immune checkpoint genes, estimate the correlation between gene expression and immune checkpoint gene expression, and generate a diagonal heat map [28]. Using a diagonal heat map, we were able to illustrate the association. As shown in the upper triangle, the *P* value and significance of the correlation are expressed in color, while the correlation coefficient is illustrated in the lower triangle. The ^*∗*^ in the graph indicates a significant correlation *P* less than 0.05, the ^*∗∗*^ represents a significant correlation *P* less than 0.01, and the ^*∗∗∗*^ indicates a significant personality *P* less than 0.001.

### 2.6. Statistical Analysis

The data were examined using the R program (Version 3.6.3, The *R* Foundation for Statistical Computing). The unpaired *t* test was applied to test the differential expression of UBE2T in cancer tissues compared to adjacent nonmalignant tissues. The log-rank test was used to evaluate the Kaplan–Meier survival curves. The Cox regression model for multivariate analysis was used to ascertain the existence of independent prognostic variables. A *P* value of less than 0.05 was used to determine a statistical significance.

## 3. Results

### 3.1. LUAD DEG Identification

**Figure 1:**
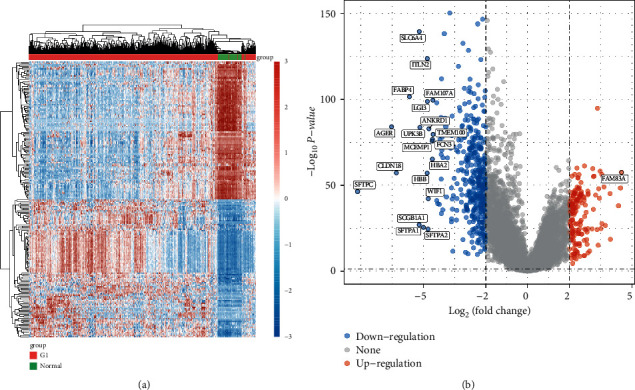
The identification of differentially expressed genes between LUAD specimens and nontumor specimens based on TCGA datasets. Both the heat map (a) and the volcano map (b) show differentially expressed genes in LUAD samples compared to normal samples. False discovery rates (FDR) are less than 0.05 and absolute log2 fold changes (log FC) are greater than 4.

This study retrospectively analyzed data from 516 LUAD samples and 59 control samples from TCGA datasets. The DEGs were analyzed using the limma package. In total, 578 DEGs were identified: 171 were significantly upregulated and 408 were significantly downregulated (Figures 1(a) and 1(b)).

### 3.2. DEGs Associated with Survival in LUAD

**Figure 2:**
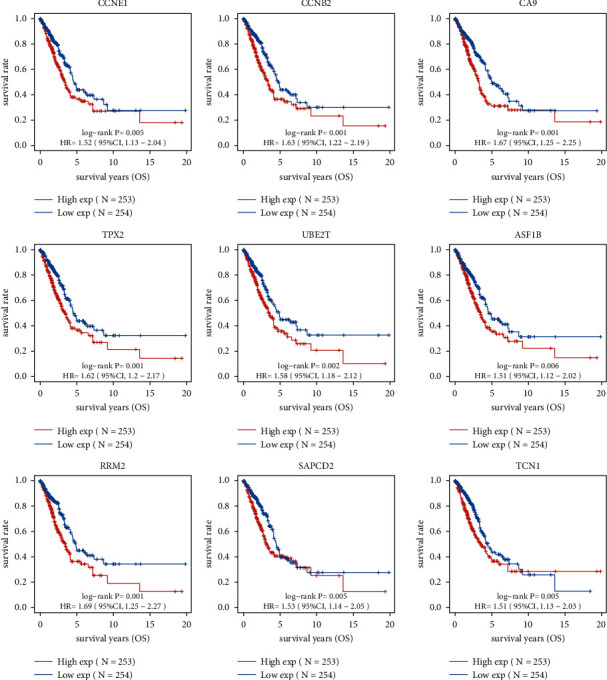
Identification of survival-related DEGs in LUAD by the use of Kaplan–Meier curves.

Then, we performed a Kaplan–Meier analysis on LUAD to screen for DEGs associated with survival in the context of survival caused by DEGs, with *P* less than 0.01. As shown in Figure 2, we identified 9 survival-related DEGs in LUAD, including ASF1B, CA9, CCNB2, CCNE1, RRM2, SAPCD2, TCN1, TPX2, and UBE2T. A PubMed search revealed that several of them have been reported in various types of tumors, including LUAD. However, no research has been conducted on the expression and function of UBE2T in LUAD. Thus, we focused on UBE2T.

### 3.3. Pan-Cancer Assays of UBE2T

**Figure 3:**
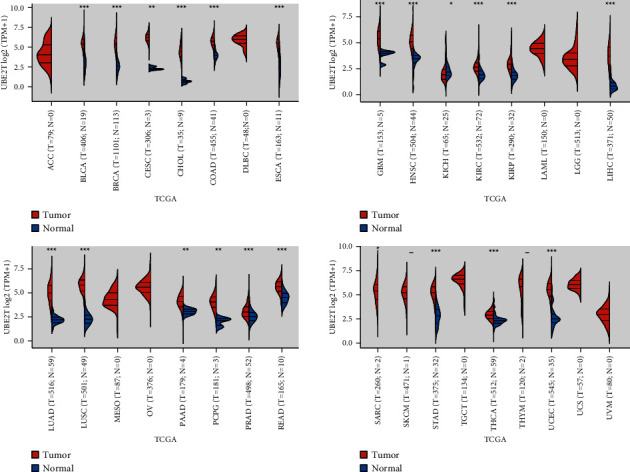
Pan-cancer expression of UBE2T between tumor tissues and normal tissues from TCGA datasets.

We examined the expression of UBE2T in several tumors and the normal tissues that bordered them to evaluate whether or not it is associated with malignancy. According to TCGA data, UBE2T mRNA expression was significantly higher in tumor tissues from the BLCA, BRCA, CESC, CHOL, COAD, ESCA, GBM, HNSC, KIRC, KIRP, LIHC, LUAD, LUSC, PAAD, PCPG, PRAD, READ, STAD, THCA, and UCEC than in normal tissues, suggesting that this molecule may play an oncogenic role in tumor progression (Figure 3). The analysis of UBE2T expression in cancer utilizing the TCGA and GTEx databases revealed a similar result as well (Figure S1). Besides, we further assessed the prognostic value of UBE2T for pan-cancer. The correlation between increased UBE2T expression and reduced overall survival in ACC, BRCA, KIRC, KIRP, LGG, LIHC, LUAD, MESO, OV, PAAD, STAD, and THYM is shown in Figure S2.

### 3.4. UBE2T in LUAD: Clinical Significance and Prognostic Value

**Figure 4:**
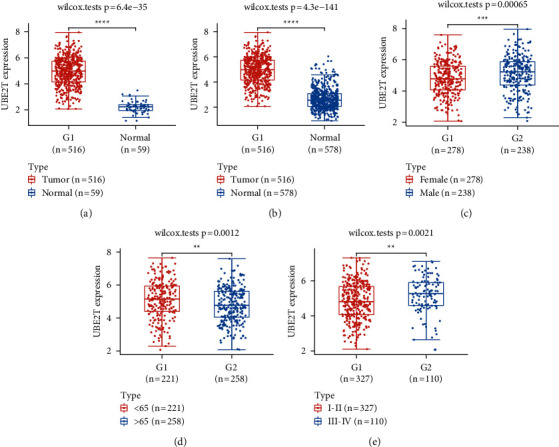
The clinical importance of the UBE2T expression in LUAD. (a and b) In LUAD samples, UBE2T expression was noticeably higher than in nontumor samples from the TCGA datasets or the TCGA and GTEx databases. (c–e) Clinical parameters that affect UBE2T expression, including (c) gender, (c) age, and (e) clinical stage.

**Figure 5:**
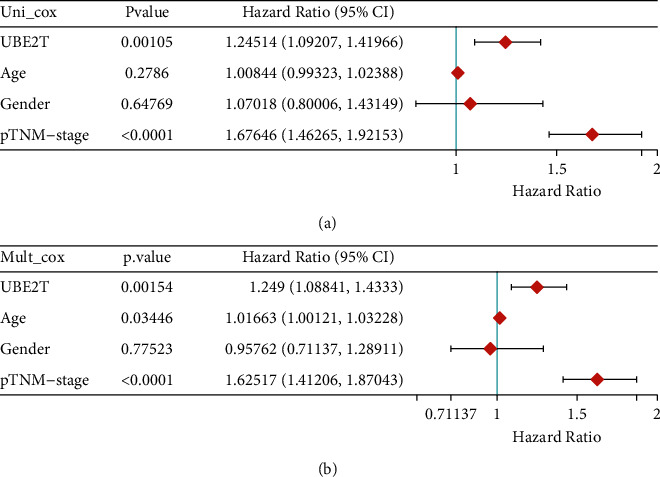
(a) Univariate and (b) multivariate analyses for overall survival of LUAD patients by Cox regression model.

First, in contrast to nontumor specimens, we discovered a clear increase in UBE2T expression in LUAD tissues (Figures 4(a) and 4(b)). The link between UBE2T expression and a number of clinical variables was then investigated. Additionally, we discovered that high UBE2T expression was associated with gender (Figure 4(c)), age (Figure 4(d)), and advanced clinical stage (Figure 4(e)). Additionally, pTNM-stage and UBE2T expression were significantly correlated, according to univariate analysis (Figure 5(a)). The UBE2T expression and pTNM-stage were shown to be independent predictive variables after multivariate data analysis (Figure 5(b)).

The degree of immune infiltration in malignancies and the expression of UBE2T are correlated.

**Figure 6:**
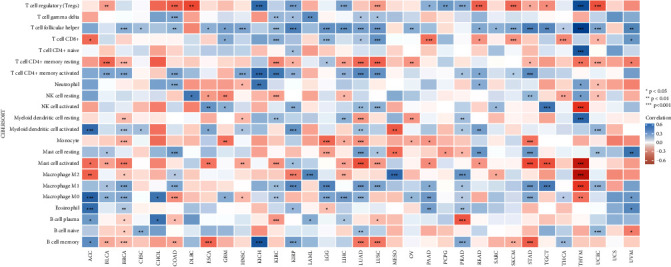
Correlation of the UBE2T expression with immune infiltration level in cancer.

As a result of tumorigenesis, the growth process is a difficult one that is accompanied by several different phenomena, such as increased proliferation, resistance to apoptosis, increased angiogenesis, and escape from immunity, among other phenomena. TME is one of them that plays an important part. TILs not only inhibited the growth of tumors but also shielded cancer cells from being destroyed, making them an important player in the fight against cancer. To look into the potential connection between UBE2T expression and immune cell infiltration, data on immune cell infiltration from two independent sources were used in a correlation study. The findings of the TIMER2 and CIBERSOR tests revealed that UBE2T was favorably linked with the amount of immune cell infiltration in the TCGA pan-cancer model (Figure S3 and Figure 6). This study's key finding was that UBE2T correlated favorably with T cell gamma delta, T cell follicular helper, T cell CD4+ memory activated, NK cell activated, macrophage M0, and B cell naive and adversely with T cell CD4+ memory resting, myeloid dendritic cell resting, mast cell activated, macrophage M2, and B cell plasma (Figure 6). Data on immune cell infiltration from three sources were consistently examined.

### 3.5. Inhibition of Immune Checkpoints by the UBE2T Expression

The relationship between the UBE2T expression and immunological checkpoint genes was examined using eight popular immune checkpoint genes.  Figure S4 presents the findings. In a variety of cancers, UBE2T expression was associated favorably with the levels of numerous immune checkpoint genes, including UVM, THCA, LIHC, LGG, KIRC, and BLCA (Figure S4). On the other hand, it was discovered that the LAG3 expression and UBE2T expression were positively associated. The expression of UBE2T and immunological checkpoint genes was examined to see whether there was a relationship between them. The research used eight popular immune checkpoint genes.

## 4. Discussions

Everyone knows that lung cancer is the sort of cancer that causes the most fatalities worldwide [29]. Over 80% of all instances of lung cancer are diagnosed in individuals with NSCLC, and about 50% of these patients have LUAD [30]. Despite improvements in medication regimens, the survival rate of individuals with LUAD remains very poor. In addition to the high-level variability of LUAD, there are a plethora of complicated etiologic factors that may make it challenging to predict the prognosis [31, 32]. Therefore, the creation of creative prognostic models is urgently required.

The TCGA database was used to get clinical and mRNA expression data from LUAD level 3 RNA seq for the present research. Then, we carried out a comparison of the differential expression between LUAD-positive samples and normal lung tissue. There were found to be 408 substantially downregulated genes and 171 significantly upregulated genes out of a total of 578 DEGs. Then, we discovered 9 DEGs in LUAD that were associated with survival. UBE2T was one among those that caught our interest. Previous studies have hypothesized that UBE2T may contribute to a variety of tumor forms. For instance, Yu et al. found that RACK1 was ubiquitinated and degraded at the lysine K172, K225, and K257 residues without the aid of an E3 ligase by UBE2T, which overactivated the Wnt/-catenin signaling pathway. This opens up a new window of possibility for particular GC patients who have abnormal Wnt/-catenin signaling [23]. Liu and his colleagues found that both the mRNA and protein levels of UBE2T were considerably greater in HCC tissues compared to nontumor tissues close to the tumor. It was also shown that UBE2T overexpression prevented hepatoma cell proliferation, colony formation, tumorigenesis, migration, and invasion, but UBE2T inhibition had the reverse effect [24]. Additionally, it was shown that the UBE2T expression was markedly increased in GBM tissues and was associated with a bad prognosis. Blocking UBE2T dramatically decreased cell invasion and migration, according to in vitro study. This was done by stabilizing GRP78 and controlling EMT [33]. UBE2T may have previously been shown to promote both autophagy and proliferation, which raises the possibility that by inhibiting this gene, lung cancer cells may not go through autophagy. It was discovered that the p53/AMPK/mTOR signaling pathway was engaged during UBE2T-mediated autophagy, proving that UBE2T induced autophagy via this mechanism. However, the prognostic value of UBE2T has not been investigated. In this study, we examined the associations between the expression of UBE2T and several clinical factors. We also found that advanced clinical stages, gender, and age were associated with higher UBE2T expression. Multivariate analysis was used to identify the p-TNM stage and UBE2T expression as independent prognostic factors. Our study showed that UBE2T has the potential to be used as a sophisticated prognostic biomarker for LUAD patients. Our findings were consistent with previous results that UBE2T may serve as a tumor promotor.

According to current thinking, TME significantly affects the clinical treatment response and prognosis of patients with malignancies [34]. This idea is supported by the advancement of precise and high-throughput technology. Immune cells that have invaded tumor patients' TMEs have been proven in an increasing number of studies to have either a pro- or an antitumorigenic function [35, 36]. A positive prognosis for LUAD patients is related to immune cell infiltration in tumors, according to Rachel and others. The TCGA database has made it feasible to gather several global gene expression profiles as well as clinical information. In accordance with our findings, UBE2T was negatively correlated with T cell CD4+ memory resting, myeloid dendritic cell resting, mast cell activated, macrophage M2, and B cell plasma, and positively correlated with T cell gamma delta, T cell follicular helper, T cell CD4+ memory activated, NK cell activated, and B cell naive. Pan-cancer tests have also shown that UBE2T is critical for TME.

The field of cancer treatment, LUAD in particular, has lately experienced a drastic upheaval as a result of considerable advancements in immunotherapy [37]. First-line pembrolizumab, an immune checkpoint inhibitor that targets PD-1, in combination with pemetrexed-carboplatin continues to demonstrate increased response and survival in advanced NSCLC in comparison to chemotherapy alone [38, 39]. Durvalumab, a human IgG1 monoclonal antibody that targets PD-L1, may prolong overall survival in Stage III non-small-cell lung cancer patients following chemoradiation [40]. Immunotherapy, however, could only benefit a tiny portion of patients if they are not picked correctly. Therefore, identifying reliable biomarkers to screen the majority of immunotherapy patients is critical. The expression of PD-L1 and TMB may serve as predictive indicators for the efficacy of ICBs, according to prior research. There are, however, restrictions to be aware of. For instance, because of the high geographical and temporal variation in the expression of PD-L1, the use of TMB is constrained since there are no uniform criteria that can be utilized to establish the cut-off value. In this study, we found that UBE2T expression was positively correlated with the expression of many immunological checkpoint genes, including UVM, THCA, LIHC, LGG, KIRC, and BLCA. However, we recently found a favorable correlation between the LAG3 expression and UBE2T expression. We infer from the aforementioned results that the immune infiltration's function in regulating UBE2T expression may have an impact on the onset and development of LUAD.

This study inevitably contains several limitations that need to be taken into account. Firstly, because the prognosis for UBE2T in this study was based on information from the TCGA databases, new clinical data are required to confirm it. Additionally, UBE2T's involvement in the mechanism that it used in LUAD samples is not currently explained by wet experimental evidence. Therefore, more works is needed to shed light on the potential connection between UBE2T and the prognosis of LUAD. We intend to investigate the impact of UBE2T on LUAD cells by in vitro invasion and migration experiments, confirm the regulatory relationship between UBE2T and EMT development, and, finally, suggest investigating the impact of UBE2T on LUAD using animal models.

## 5. Conclusions

LUAD had an increased expression of UBE2T and its expression was significantly correlated with variables such as gender, age, and advanced clinical stage. Patients with high levels of UBE2T expression exhibited significantly shorter overall survival rates, and UBE2T could be used as a biomarker for LUAD prognosis. These findings not only offered crucial cues for the identification of novel treatment targets in LUAD but they also established a framework for the investigation of potential UBE2T pathways in LUAD.

## Data Availability

The data used to support the findings of this study are included within the article.

## Supplementary Materials

Supplementary Materials Figure S1 displays the pan-cancer expression of UBE2T in tumor tissues out from the TCGA database as well as in normal tissues from the TCGA and GTEx datasets. Figure S2 The predictive relevance of UBE2T in the above tumor types was assessed using univariate analysis from the TCGA database. The median UBE2T value was chosen as the cut-off value for each tumor. Figure S3 UBE2T expression and immune infiltration levels in malignancies were correlated using TIMER2. Figure S4 Immune checkpoints and UBE2T expression are related. Table S1 The extension of tumor abbreviations.
