# Supplementary material for: Prognostic Value of UBE2T and Its Correlation with Immune Infiltrates in Lung Adenocarcinoma
Source: J Oncol. 2022 Sep 20;2022:5244820. doi: 10.1155/2022/5244820 (PMC9553516; doi:10.1155/2022/5244820)
Supplement: Supplementary Materials — Figure S1 displays the pan-cancer expression of UBE2T in tumor tissues out from the TCGA database as well as in normal tissues from the TCGA and GTEx datasets. Figure S2 The predictive relevance of UBE2T in the above tumor types was assessed using univariate analysis from the TCGA database. The median UBE2T value was chosen as the cut-off value for each tumor. Figure S3 UBE2T expression and immune infiltration levels in malignancies were correlated using TIMER2. Figure S4 Immune checkpoints and UBE2T expression are related. Table S1 The extension of tumor abbreviations. [file 5244820.f1.zip › Table S1.docx]

Table S1 The extension of tumor abbreviations

| **TCGA** | **Detail** |
| --- | --- |
| ACC | Adrenocortical carcinoma |
| BLCA | Bladder Urothelial Carcinoma |
| BRCA | Breast invasive carcinoma |
| CESC | Cervical squamous cell carcinoma and endocervical adenocarcinoma |
| CHOL | Cholangio carcinoma |
| COAD | Colon adenocarcinoma |
| DLBC | Lymphoid Neoplasm Diffuse Large B-cell Lymphoma |
| ESCA | Esophageal carcinoma |
| GBM | Glioblastoma multiforme |
| HNSC | Head and Neck squamous cell carcinoma |
| KICH | Kidney Chromophobe |
| KIRC | Kidney renal clear cell carcinoma |
| KIRP | Kidney renal papillary cell carcinoma |
| LAML | Acute Myeloid Leukemia |
| LGG | Brain Lower Grade Glioma |
| LIHC | Liver hepatocellular carcinoma |
| LUAD | Lung adenocarcinoma |
| LUSC | Lung squamous cell carcinoma |
| MESO | Mesothelioma |
| OV | Ovarian serous cystadenocarcinoma |
| PAAD | Pancreatic adenocarcinoma |
| PCPG | Pheochromocytoma and Paraganglioma |
| PRAD | Prostate adenocarcinoma |
| READ | Rectum adenocarcinoma |
| SARC | Sarcoma |
| SKCM | Skin Cutaneous Melanoma |
| STAD | Stomach adenocarcinoma |
| TGCT | Testicular Germ Cell Tumors |
| THCA | Thyroid carcinoma |
| THYM | Thymoma |
| UCEC | Uterine Corpus Endometrial Carcinoma |
| UCS | Uterine Carcinosarcoma |
| UVM | Uveal Melanoma |
