# Supplementary figures and images for: Prognostic Value of UBE2T and Its Correlation with Immune Infiltrates in Lung Adenocarcinoma
Source: J Oncol. 2022 Sep 20;2022:5244820. doi: 10.1155/2022/5244820 (PMC9553516; doi:10.1155/2022/5244820)

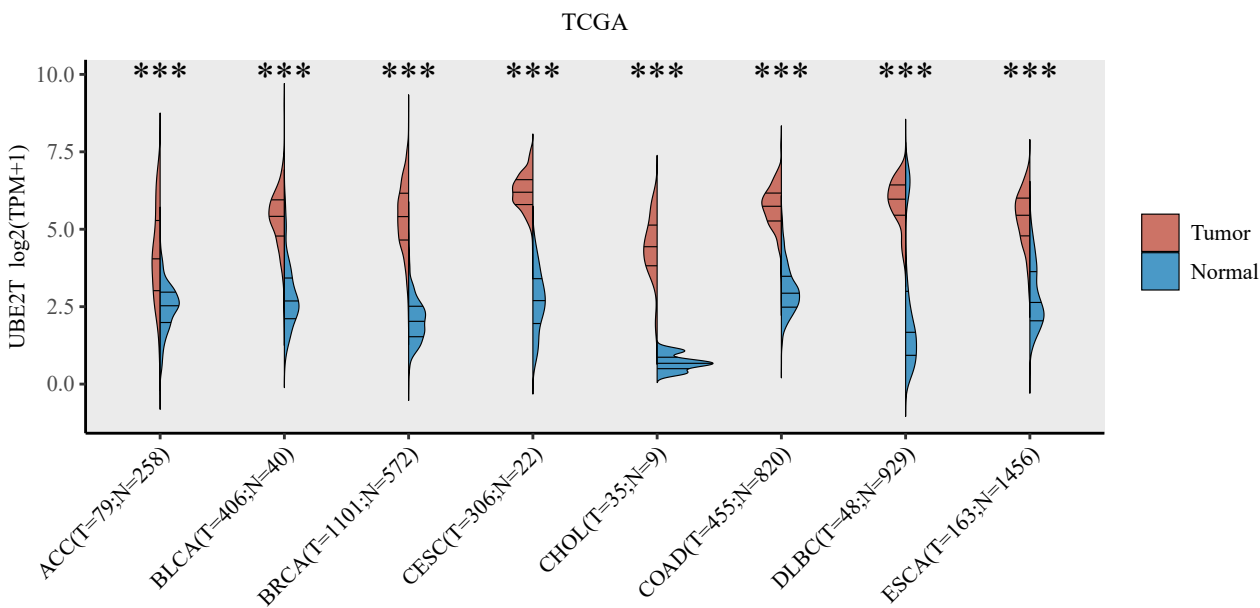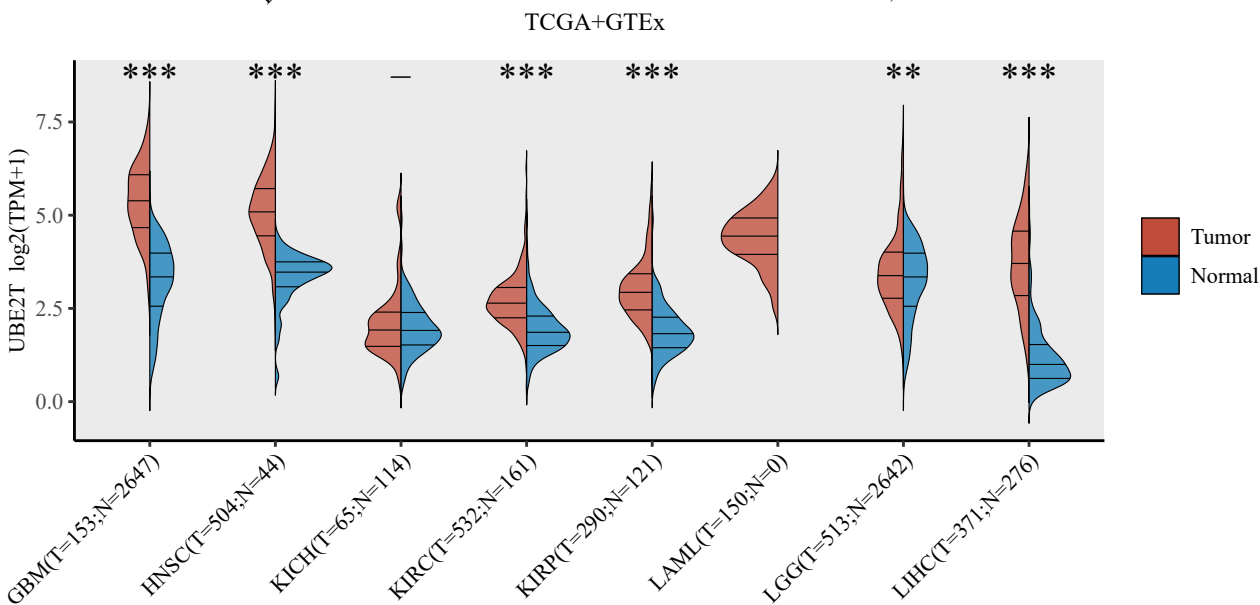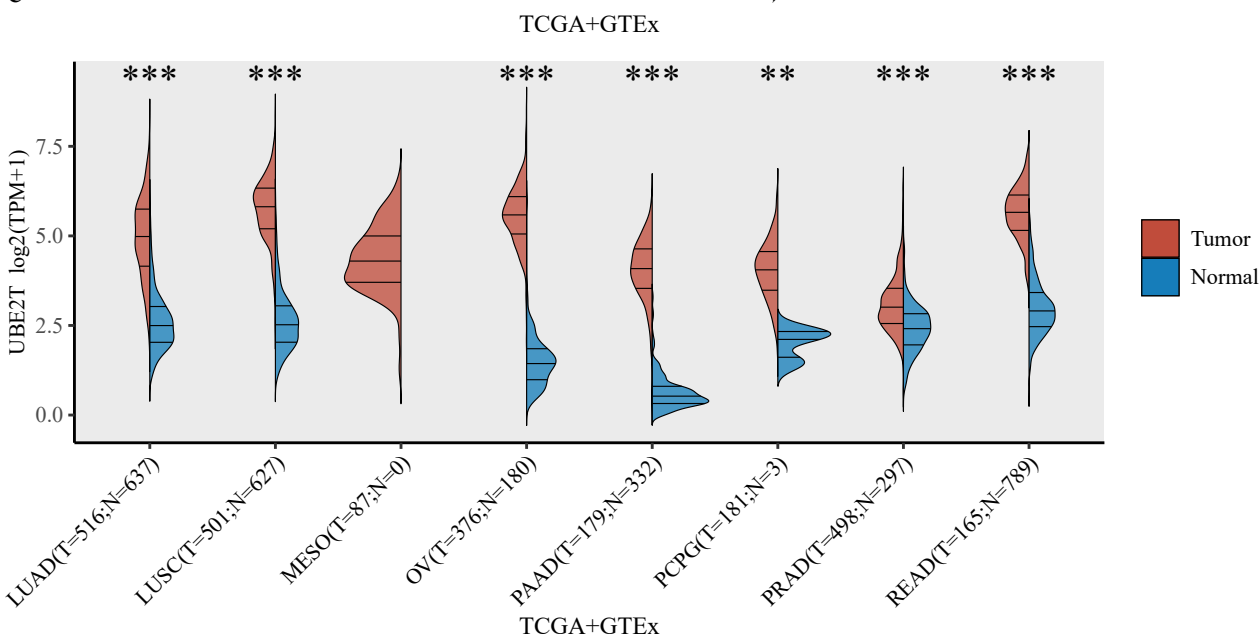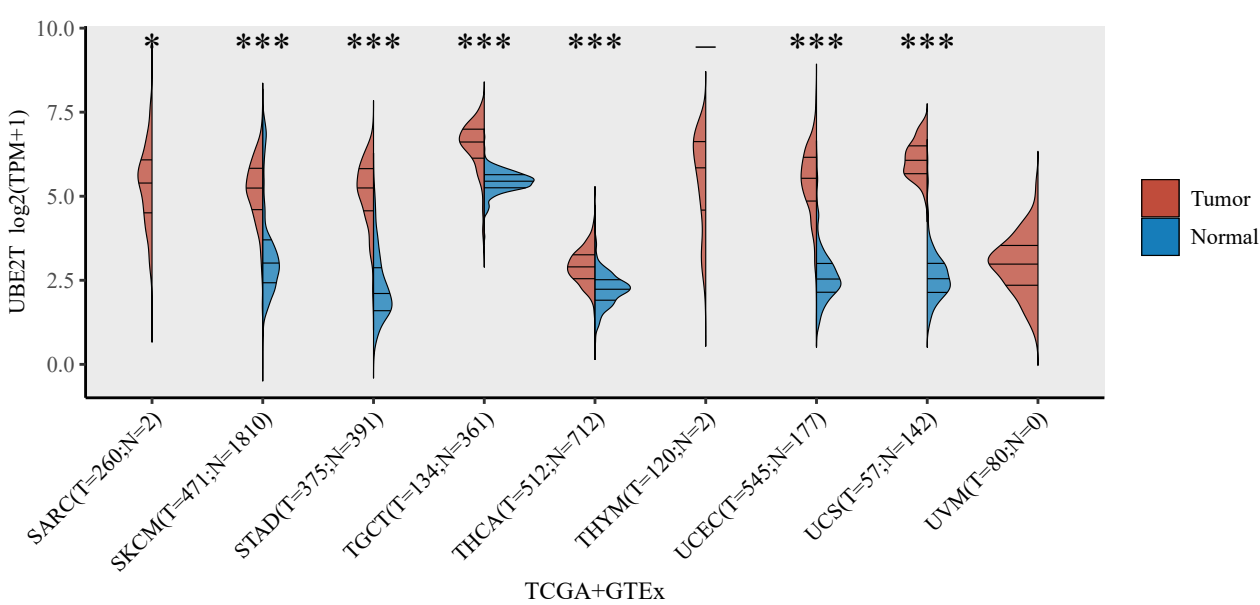

Supplement: Supplementary Materials — Figure S1 displays the pan-cancer expression of UBE2T in tumor tissues out from the TCGA database as well as in normal tissues from the TCGA and GTEx datasets. Figure S2 The predictive relevance of UBE2T in the above tumor types was assessed using univariate analysis from the TCGA database. The median UBE2T value was chosen as the cut-off value for each tumor. Figure S3 UBE2T expression and immune infiltration levels in malignancies were correlated using TIMER2. Figure S4 Immune checkpoints and UBE2T expression are related. Table S1 The extension of tumor abbreviations. [file 5244820.f1.zip › Figure S1.pdf]

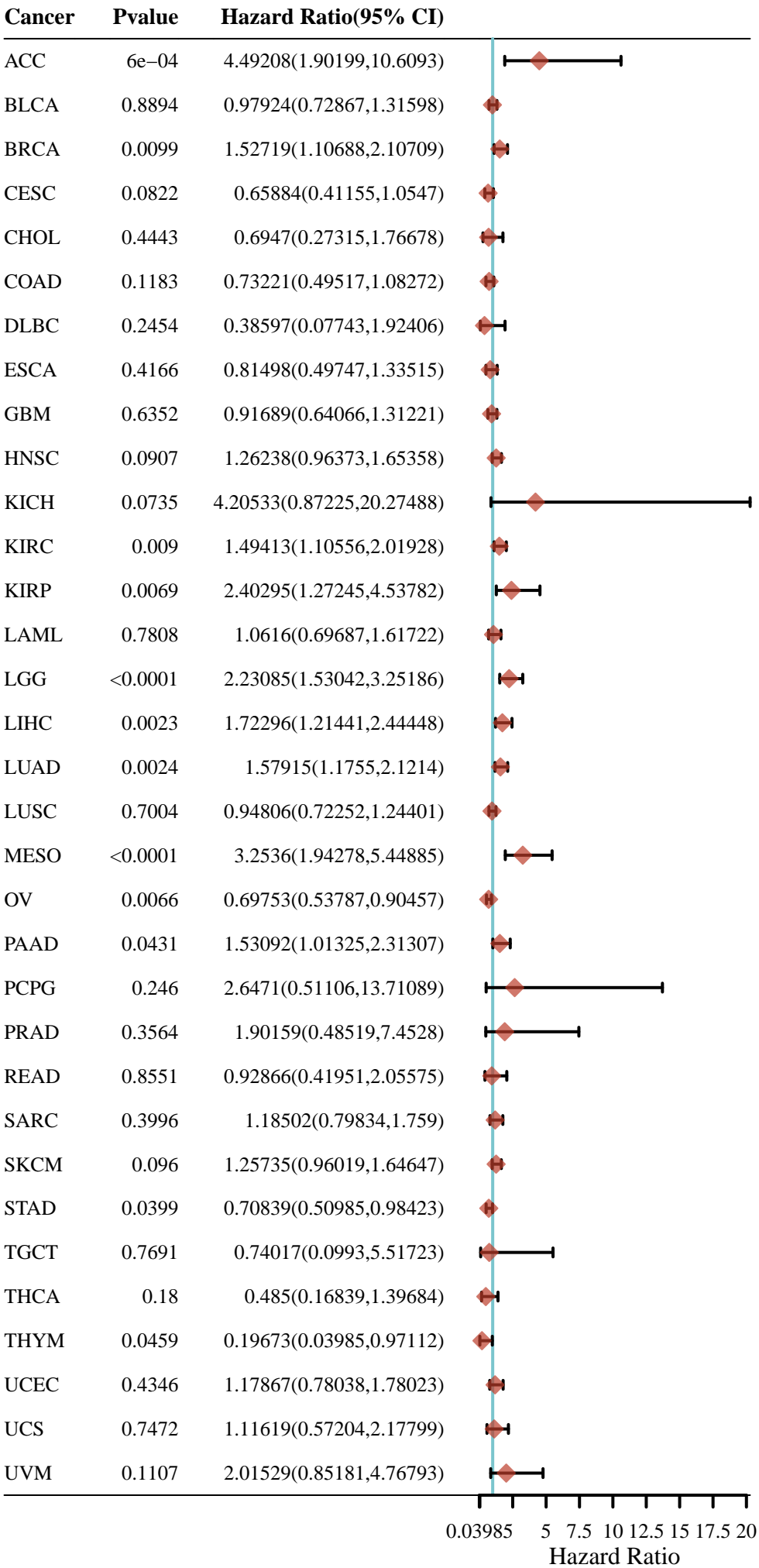

Supplement: Supplementary Materials — Figure S1 displays the pan-cancer expression of UBE2T in tumor tissues out from the TCGA database as well as in normal tissues from the TCGA and GTEx datasets. Figure S2 The predictive relevance of UBE2T in the above tumor types was assessed using univariate analysis from the TCGA database. The median UBE2T value was chosen as the cut-off value for each tumor. Figure S3 UBE2T expression and immune infiltration levels in malignancies were correlated using TIMER2. Figure S4 Immune checkpoints and UBE2T expression are related. Table S1 The extension of tumor abbreviations. [file 5244820.f1.zip › Figure S2.pdf]

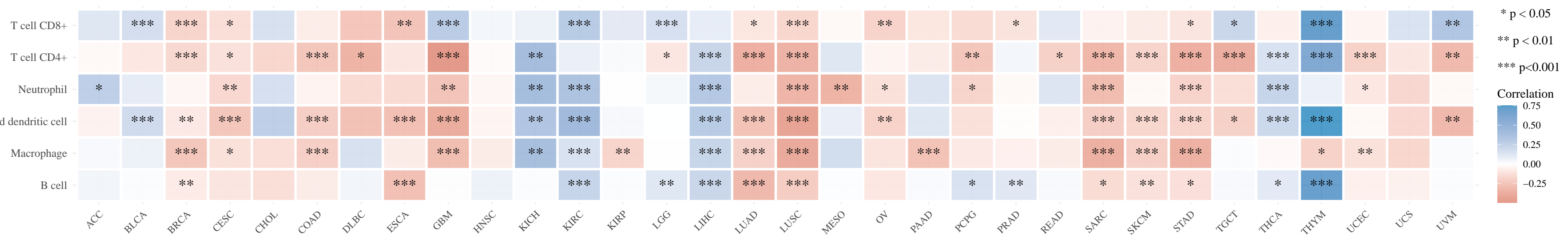

Supplement: Supplementary Materials — Figure S1 displays the pan-cancer expression of UBE2T in tumor tissues out from the TCGA database as well as in normal tissues from the TCGA and GTEx datasets. Figure S2 The predictive relevance of UBE2T in the above tumor types was assessed using univariate analysis from the TCGA database. The median UBE2T value was chosen as the cut-off value for each tumor. Figure S3 UBE2T expression and immune infiltration levels in malignancies were correlated using TIMER2. Figure S4 Immune checkpoints and UBE2T expression are related. Table S1 The extension of tumor abbreviations. [file 5244820.f1.zip › Figure S3.pdf]

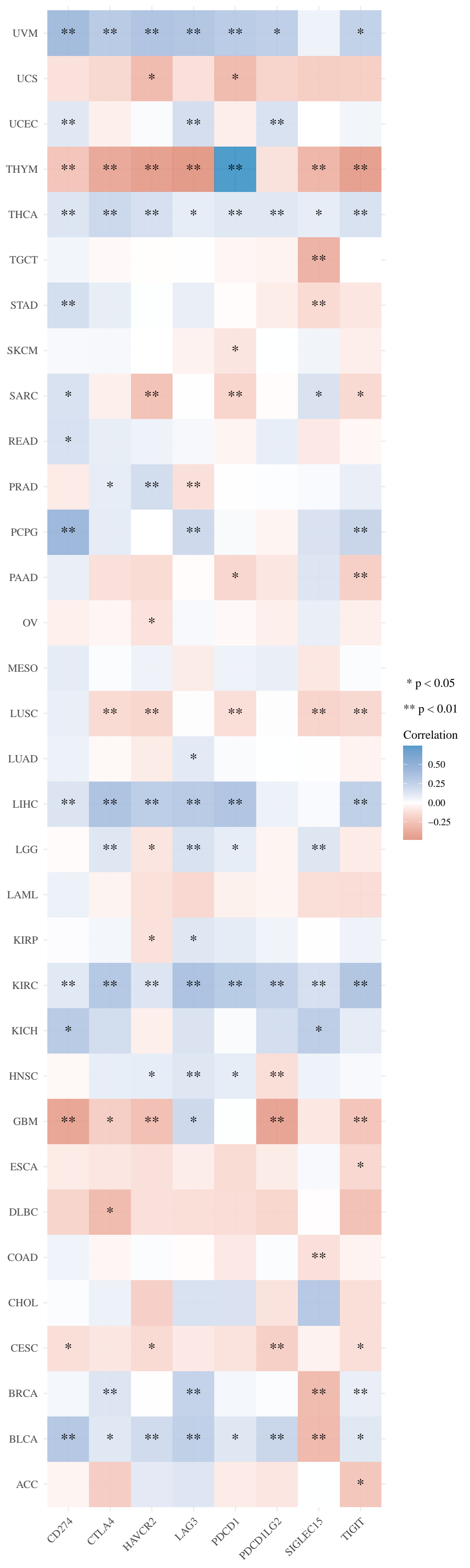

Supplement: Supplementary Materials — Figure S1 displays the pan-cancer expression of UBE2T in tumor tissues out from the TCGA database as well as in normal tissues from the TCGA and GTEx datasets. Figure S2 The predictive relevance of UBE2T in the above tumor types was assessed using univariate analysis from the TCGA database. The median UBE2T value was chosen as the cut-off value for each tumor. Figure S3 UBE2T expression and immune infiltration levels in malignancies were correlated using TIMER2. Figure S4 Immune checkpoints and UBE2T expression are related. Table S1 The extension of tumor abbreviations. [file 5244820.f1.zip › Figure S4.pdf]
